# A Microfluidic Device to Sort Cells Based on Dynamic Response to a Stimulus

**DOI:** 10.1371/journal.pone.0078261

**Published:** 2013-11-08

**Authors:** Swee Jin Tan, Michelle Z. L. Kee, Ajay Sriram Mathuru, William F. Burkholder, Suresh J. Jesuthasan

**Affiliations:** 1 Microfluidics Systems Biology Lab, Institute of Molecular and Cell Biology, Singapore, Singapore; 2 Neuroscience and Behavioral Disorders Program, Duke-National University of Singapore Graduate Medical School, Singapore, Singapore; 3 Neural Circuitry and Behavior Lab, Institute of Molecular and Cell Biology, Singapore, Singapore; 4 Department of Physiology, National University of Singapore, Singapore, Singapore; Monell Chemical Senses Center, United States of America

## Abstract

Single cell techniques permit the analysis of cellular properties that are obscured by studying the average behavior of cell populations. One way to determine how gene expression contributes to phenotypic differences among cells is to combine functional analysis with transcriptional profiling of single cells. Here we describe a microfluidic device for monitoring the responses of single cells to a ligand and then collecting cells of interest for transcriptional profiling or other assays. As a test, cells from the olfactory epithelium of zebrafish were screened by calcium imaging to identify sensory neurons that were responsive to the odorant L-lysine. Single cells were subsequently recovered for transcriptional profiling by qRT-PCR. Responsive cells all expressed *TRPC2* but not *OMP*, consistent with known properties of amino-acid sensitive olfactory neurons. The device can be adapted for other areas in biology where there is a need to sort and analyze cells based on their signaling responses.

## Introduction

All biological systems, from multi-species microbial consortia to the adaptive immune system, cancer stem cells and the brain, are characterized by diversity at the single cell level [1], [2], [3]. This richness can result from normal developmental processes, e.g. Rag1/Rag2 mediated recombination [4] and gene expression changes following signaling events, or can be induced by environmental stressors that cause chromosomal changes [5] as well as the inherent stochasticity of biochemical reactions [6]. Variation at the single cell level may be deleterious as in the case of nervous system disorders [7], but can increase fitness and improve performance of biological systems, for example by broadening the range of signal detection.

To understand the molecular basis of diversity in signaling, it would be useful to have a method that couples molecular analysis with an assessment of signaling at the single cell level. Several techniques for characterizing the genome, transcriptome and metabolome of single cells are currently available [8], [9], [10]. Additionally, several microfluidic-based methods for isolating single cells have been devised. These include magnetic separation [11], electro-osmotic-based sorting [12], piezoelectric actuation in a continuous flow [13], and a dynamic array of traps that utilizes dielectrophoretic fields to capture and selectively recover multiple single cells [14]. However, these techniques introduce a significant external field, which can interfere with measurements of cellular responses and affect cell viability. Other sorting techniques that are considered gentle to cells include separation through deterministic lateral displacement [15], [16] or alteration of laminar flow characteristics in micro flows [17], [18]. All of these techniques, however, do not allow the introduction of an external stimulant for sorting cells based on ligand-induced responses.

Here, we describe a device that allows monitoring of signaling in living cells, followed by sorting for molecular characterization. We test the device using cells from the olfactory system of the zebrafish. Like other vertebrates, the zebrafish has a large repertoire of odorant receptors [19], [20], [21]. Each olfactory sensory neuron expresses one or a few receptors [22], but each receptor can bind several ligands. The animal is able to recognize odorants occupying a large chemical space because of the diversity of the receptors [23]. One method that has been used to identify olfactory sensory neurons that respond to a particular ligand is to attach a group of neurons to a cover slip, flow the odorant over the neurons and monitor increase in intracellular calcium levels [24]. A responding cell can then be picked using a micromanipulator and molecularly characterized. The device described here performs a similar procedure, but on cells in suspension, avoiding difficulties that arise from temporarily attaching cells that have to be subjected to a flow. We show that olfactory sensory neurons in suspension are able to respond to an odorant, and that the device can accurately sort cells responsive to the stimulus.

The modular nature of the system allows additional functionality, such as cell lysis or sample processing (mRNA extraction and cDNA synthesis) to be implemented on-chip, with the potential to increase the efficiency at which cells can be characterized. We propose that this device is adaptable to other biological systems where the sorting of cells based on their dynamic response to a stimulus is an important step in understanding complexity.

## Materials and Methods

### Microdevice fabrication procedures

**Figure 1:**
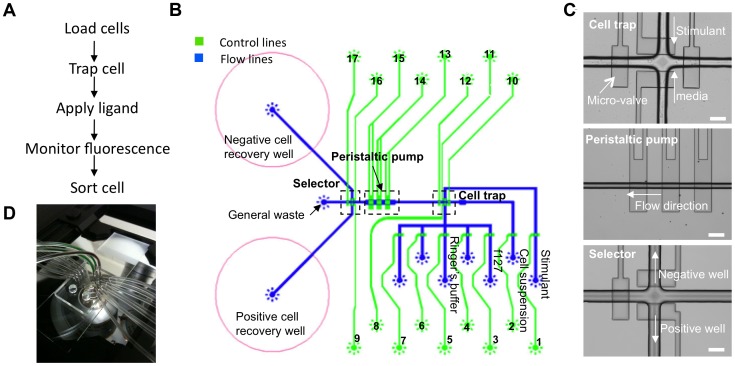
A microfluidic device for sorting cells based on dynamic responses. (A) Outline of the operations performed using the chip. (B) Schematic of the device. The main components are the peristaltic pump, cell trap and selection wells. The numbered circles represent points for insertion of 23 gauge needle connectors. Blue lines represent flow lines, which are located in the lower layer of the device (Figure S1 in File S1), while green lines are control lines, which are located in the upper layer of the device (Figure S2 in File S1). Cells are introduced via a flow line, and their movement is regulated by the control lines that operate the peristaltic pump and seal the cell-trap by actuating push-down valves. The stimulus is then delivered via another flow line. After this, cells are directed to one of two large wells for recovery. Flow lines for Pluronic-F127 and Ringer's buffer are used to prepare the chip. Other flow lines are available for introduction of multiple stimuli to the cell trap. (C) Different components in the system for capturing single cells and introducing stimulants (cell trap), controlling the movement of cells (peristaltic pump), and cell sorting (selector), as seen in a finished device. Scale bar represents 100 µm. (D) An operational device mounted on a microscope. The tygon tubes are used to provide control and introduce reagents.

The microdevice was created using soft lithography as summarized in Figure S1 in File S1 using standard methods that have been described previously [25], [26], [27]. The photo mask was drawn in AutoCAD 2011 (Autodesk, Inc., San Rafael, CA, USA) and produced on glass with critical dimensions of 5 µm (IMRE, Singapore). The AutoCAD file is available in the accompanying File S2, and additional information regarding the file format and general design considerations for PDMS-based large-scale integrated microfluidic devices can be found at http://www.stanford.edu/group/foundry/services/mask_design_rules.html). The master control mould (for the control lines shown in green in Figure 1B) was fabricated by spin coating (2700 rpm, 30 s) SU-8 2025 (MicroChem Corp., Newton, MA, USA) on a 4-inch silicon wafer achieving a 23 µm depth. The flow mould (for sample and reagent flow lines shown in blue in Figure 1B) was fabricated by spin coating (1700 rpm, 60 s) SPR 220-7.0 (Rohm and Hass, Midland, MI, USA) on another 4-inch silicon substrate yielding a 15 µm thickness. The flow mould was further subjected to a heat reflow process by incubating at 190°C for 30 minutes followed by a slow ramp down to room temperature. This changes the photoresist profile from a rectangular cross-section to a curved surface that ensures that the microfluidic valves are closed completely during actuation [28].

Using these moulds, the control and flow layers of the device were cast out of polydimethylsiloxane (PDMS) (Sylgard 184, Dow Corning Corp., Midland, MI, USA) and manually assembled under a stereoscope (Nikon, Japan) essentially as described [28], [29]. Base to curer ratios of 5∶1 and 20∶1 were used for the control and flow layers respectively. The larger ratio used for the flow layer produced a fairly flexible membrane that bowed downwards easily when a positive pressure was applied in the control fluidic lines, whereas the smaller ratio used for the control layer resulted in a harder material that increased the overall rigidity of the device. The control and flow layers were bonded to each other by first partially curing each layer at 80°C (20 min for the control layer and 25 min for the flow layer) [30] and then bringing them together and heating at 80°C for one hour. After this, control fluidic ports (holes) and cell recovery wells were punched with a gauge 23 needle and 5 mm biopsy punch respectively. The finished device was then bonded onto a cleaned glass substrate by incubating at 80°C overnight.

### Device preparation and operation

For pneumatic control of the device, the fluidic control ports (Figure 1B) were connected to pneumatic solenoid valves (Pneumadyne Inc., USA) using 23 gauge needle connectors attached to tygon tubing. The valves were linked to pressure sources that provided constant pressures of 5 and 20 psi and were operated electronically using a custom-built USB-based electronic controller unit interfaced to a PC (see Figure S2 in File S1 for a schematic of the experimental setup; detailed instructions for assembling the electronic controller are available at http://www.stanford.edu/group/foundry/testing/own_controller.html). Valve operation was automated using scripts written in NI Labview 2009 (National Instruments Corp., Austin, TX, USA; an executable file is available in File S3). Before use, control and flow lines within the microdevice were filled with buffer utilizing dead-end filling and pressure driven flow [28], [29]. The buffer in the flow lines was then replaced with 1% Pluronic F-127 (a surfactant that ensures cells do not adhere to the surface) and incubated for 1 hr. These steps for preparing the chip took approximately 75 min.

To operate the device after coating with Pluronic F-127, the flow channels were flushed for 5 min at 5 psi with Ringer's buffer (116 mM NaCl, 2.9 mM KCl, 1.8 mM CaCl_2_, 5 mM HEPES, pH 7.2) to remove any traces of the surfactant before cell loading. Introduction of the stimulant to the cell capture chamber shown in Figure 1C was achieved by opening micro-valves 11, 12 and 17 (Figure 1B). Subsequently, micro-valve 11 was closed and micro-valve 8 was opened to remove excess stimulant in the system by flushing with buffer, directing the flow to the waste outlet. The device was now ready for the introduction of cells. Cell suspensions (50–100 µl) were loaded and directed to the cell trap by the on-chip peristaltic pump. The pump was operated by sequential actuation of the valves in the pattern 101, 100, 110, 010, 011, 001 where 1 represents an activated valve and 0 shows an open valve. Although a wide range of operating frequencies are possible (up to 100 Hz [28]), a frequency of 1.67 Hz was found to allow manual tracking of objects within the cell-trap, with a flow rate was approximately 200 µm/s. When a single cell was observed to enter the cell trap region, the peristaltic pump was stopped and the micro-valves surrounding the cell trap were closed. A baseline fluorescence image of the trapped cell was then taken. Micro-valve 11 was subsequently opened for 15 s to expose the trapped cell to stimulant. During this phase, fluorescence images were taken at intervals of 1–3 s and the maxima fluorescence intensity was recorded. Positive cells (registering an intensity change of greater than 2%) were directed to the positive collection well. Otherwise cells were discarded or sent to the negative collection well. Flow from the stimulation line into the cell trap was tested with a green food dye (Tartrazine E102, Brilliant Blue E133, Carmosine E122; Star Brand, Singapore). Intensity was measured using imageJ.

### Preparation of samples

Experiments were approved by the Institutional Animal Care and Use Committee of the Biological Resource Centre at Biopolis (#120730). Olfactory epithelium cells isolation and dissociation methods were modified from Corotto *et al.* [31]. Briefly, AB wild type or TRPC2: Venus [32] zebrafish (a gift of Y. Yoshihara from the RIKEN Brain Science Institute) were euthanized in ice-water [33]. Olfactory rosettes were isolated in calcium-free Ringer's buffer (116 mM NaCl, 2.9 mM KCl and 5 mM HEPES, pH 7.2). Rosettes were suspended in 100 µl calcium-free Ringer's with 2 mU of Papain (Worthington Biochemical Corporation, Lakewood, NJ, USA) and 2 units of DNaseI, and incubated for 15 mins at 37°C with shaking at 300 rpm. After replacing the solution with standard Ringer's buffer, cells were dissociated into suspension by trituration to minimize cell clumping.

For cell viability studies, trypan blue (Life Technologies, USA) was added to the cell suspension in a 1∶1 ratio with stock solution (0.4%). For on-chip studies, the dye was introduced via the stimulation inlet.

For the ionophore or odorant stimulation experiments, 2 µM of a fluorescent calcium indicator, Fluo 4-AM or Rhod 2-AM (Life Technologies Corp., USA), was added to the cell suspension after extraction and incubated for 15 mins at room temperature following manufacturer's recommendation. This led to a uniform labelling of the cell. To remove excess dye, cells were collected by centrifugation, washed and resuspended in 100 µl Ringer's buffer. 5 µM A23187 (Life Technologies Corp., USA) was used to induce calcium influx.

### Image acquisition

The microdevice was mounted on the Olympus inverted microscope (IX71, Olympus Corp., Japan) and a 20× or 40× objective was used to image cells in the cell trap. Bright field and fluorescence images were captured using a CoolSNAP EZ camera (Photometrics, Tucson, AZ, USA) and filter sets U-MWB2 (Ex: 460–490 nm/Em: 520 nm; YFP, Fluo 4-AM) and U-MWG2 (Ex: 510–560 nm/Em: 590 nm; Rhod 2-AM) from Olympus Singapore Pte Ltd (Singapore). Excitation was provided by a mercury arc lamp (100 W). Measurement of fluorescence intensities during the experiment was done via ImageJ software [34]. Background subtraction and segmentation were applied to every image frame to obtain the average intensity of the whole cell. The use of average intensity (i.e. total intensity/area) compensated for changes in total intensity that may occur due to slight variations in focal plane. The ratio of the fluorescence intensity change (ΔF) to the baseline fluorescence value before stimulation (F_0_) was calculated for each frame (ΔF/F_0_).

### qRT-PCR of single cells

Single cell qRT-PCR was performed to ascertain the expression profiles of the genes TRPC2, OMP, EF1α, B2M, and β-Actin (primer sets used for qRT-PCR are listed in Table S1 in File S1). The protocol was adapted from Dalerba *et al.* [35]. Briefly, after sorting a cell and verifying under bright-field microscopy that a single cell was present in the recovery well, 5 µl of CellsDirect (Life technologies, USA) premixed with SuperaseIn RNase-inhibitor at a ratio of 50∶1 (v/v; 2 U of SuperaseIn RNase-inhibitor per sample) was added to the well and the cell was transferred to a PCR tube on ice. Cells were lysed at 75°C for 10 min and first-strand cDNA synthesis was performed using the SuperScript III reverse transcriptase kit (Life Technologies Corp, USA) following the manufacturer's instructions. qRT-PCR was performed on a Stratagene MX2005P (Agilent Technologies Inc, Santa Clara, CA, USA). Each reaction contained 10% of the total cDNA synthesized from each cell, Maxima SYBR Green qPCR master mix (Fermentas Inc., USA) and 0.3 µM of the forward and reverse primers (Table S1 in File S1). Amplification was performed with the thermal cycling conditions: 1 cycle of 95°C for 10 mins; a preamplification step consisting of 20 cycles of 95°C for 15 s, 54°C for 30 s and 72°C for 30 s, followed by 40 cycles using the same thermocycling parameters. Melting curve analysis was performed at the completion of each run. Fluorescence signals for two channels using SYBR Green (495 nm) and ROX (535 nm) were recovered. The delta-Ct values [(β-Actin Ct)-(OMP, E1α, or B2M Ct)] were then plotted.

## Results

### Microfluidic device design

A schematic representation and overview of the microfluidic chip is depicted in Figure 1. The device contains three main components to achieve single cell manipulation, monitoring, and cell sorting: a cell trap, an on-chip peristaltic pump, and a selector to sort cells to either a “positive” selection well or a “negative” selection well (Figure 1B, C). Fabricated from PDMS (Figure S1 in File S1), the chip is optically transparent and compatible with most existing inverted and upright microscopes. This minimizes start-up costs while permitting real-time visualization of cells by fluorescence microscopy or other methods before and after exposure to one or more treatment conditions.

The cell trap is a region enclosed by four micro-valves where a single cell enters and is held in place by actuating (closing) the four valves. The trapped cell is exposed to a predetermined stimulus by controlling the micro-valve attached to the stimulant flow line. With this layout, we can precisely control the exposure time and synchronize stimulation with imaging to measure the instantaneous changes within the isolated cell via fluorescence microscopy. The cell can then be released to allow the entry of a new cell. This provides robust and systematic interrogation of single cells.

The on-chip peristaltic pump provides a gentle yet precise means to direct cells to the cell trap from the inlet where cells are loaded onto the chip by pipetting. The peristaltic pump consists of three push-down micro-valves (200 µm×100 µm) [28], [29] that are arranged in series and actuated sequentially. The speed that cells traverse through the device correlates with the frequency at which the micro-valves are actuated, and a frequency of 1.67 Hz, was determined empirically to enable manual tracking of objects. The flow direction of cells in the micro-channel can also be manipulated to correctly position cells in the trap [29]. In contrast to pressure driven flow, the use of an on-chip peristaltic pump provides greater flexibility and accuracy and enables a limiting number of cells to be examined with minimal loss.

The selector unit on the chip is the component that allows the user to direct a cell to one of the two collection wells based on the phenotype observed after stimulation in the cell trap. To facilitate cell recovery, retrieval is performed by pressure driven flow at 5 psi into the designated collection well shown in Figure 1C. This enhances the turnover rate for screening and sorting cells. The recovered cell can be visualized at the collection well and removed by pipetting out of the well.

### Microdevice performance

**Figure 2:**
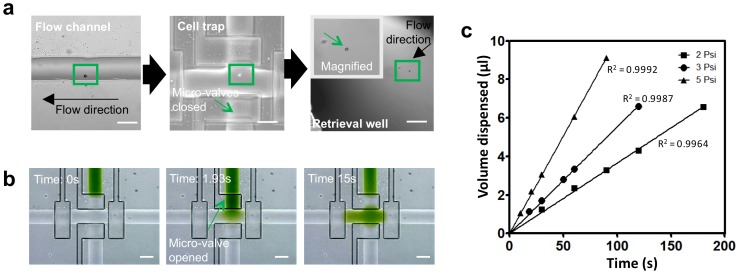
Operational characteristics for stimulation and cell recovery. (A) Tracking of a single cell (indicated by the green box) traversing through the device. The cell can be visualized within the flow line, in the cell trap and in the recovery well. (B) Visualization of the stimulation procedure using a green dye. The dye is initially in the stimulation line, and fills the entire cell trap chamber within 15 seconds. (C) Relationship between volume dispensed during the cell recovery process and the operational pressure. Scale bar represents 100 µm.

Different components in the microfluidic device work in tandem to accomplish precise control and active stimulation before sorting each incoming cell. Control of the process is semi-automated through custom scripts in Labview, which eases device operation for cell interrogation and sorting. Figure 2A shows the tracking of a cell throughout the entire process, confirming the ability of the device to capture and recover single cells.

We characterized how the relative concentration of a stimulant changed over time after injection from the stimulation flow line into the cell trap using a green food dye (Figure 2B). To ensure efficient stimulant delivery without displacing the isolated cell, a positive pressure was kept in the trap during stimulant injection. The deformability of the PDMS polymer used to fabricate the device allows the cell trap to expand to accommodate the injected volume of stimulant. As shown in Figure 2B, the entire cell-trapping chamber with dimensions of 100 µm by 200 µm was filled with green color dye within a span of 15 s. The stimulant-connecting micro-valve was then closed to allow the space to equilibrate. By measuring intensity, the cell trap was found to contain approximately 40% of the concentration of the dye in the stimulant line. During the steps used to recover cells from the trap, the dye was flushed completely from the trap and replaced by cell suspension buffer from the flow line leading into the trap. To prevent cross-contamination during the next cell stimulation, a portion of the green dye from the stimulus line was flushed out to waste.

For determining the optimal cell input concentration, we utilized varying concentrations of 10 µm polystyrene beads (Life Technologies Corp., USA) to simulate cells and monitored the number of beads captured within the cell trap during each cycle of trapping and recovery. The probability of capturing two or more beads in the trap was very low (<2%) for samples ranging from 2,000 beads per ml to 2,000,000 beads per ml but sharply increased at higher concentrations (Table S2 in File S1). The number of beads that could be trapped and recovered per minute increased with concentration (Table S2 in File S1). Thus, the optimal input concentration for the pumping condition used (1.67 Hz) was around 2 million beads per ml, which allowed an average of 53 beads to pass through the cell trapping region per minute, corresponding to approximately 1 cell per second into the trap. In cases when two or more cells are trapped, the cells can be discarded to the waste outlet utilizing the selector (Figure 1B).

Central to the operation of the device is the cell sorting and recovery capability. To ensure a reliable retrieval, the volume of media dispensed by the system during cell ejection was measured as a function of input pressure. As illustrated in Figure 2C, the volume disbursed by the system is a function of both the input pressure and the amount of time given for cell ejection. The relationship at each operating condition over time is linear since the fluidic resistance in the flow line remains the same at different controlling pressures. Over the range of conditions tested, we derived the relationship as denoted in the equation:
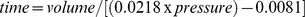
where time (seconds) denotes the duration required to achieve the intended volume dispensed (microliter) at the designated operating pressure (psi). The ability to recover cells in a relatively constant volume of buffer is very beneficial for downstream applications such as single cell RNA isolation for qPCR.

### Cell viability and sorting efficiency

The effect of the device on cell survival was examined using cells isolated from the zebrafish olfactory epithelium. Cell viability was assayed by incubation with trypan blue , a dye that is taken up only by dying cells [36]. Immediately after dissociation, cell viability was estimated at 87% (n = 77 cells; counted on a heamocytometer). Of a different batch of 60 cells that were loaded onto the device, trapped, and exposed to trypan blue administered via the stimulus inlet. 9 dead cells were recorded over a period of 2 hrs (85% viable). A count of the remaining cell suspension after 2 hrs yielded 84% viable cells. Thus, the device had minimal impact on cell viability.

As a test of sorting efficiency, samples of unlabeled and fluorescently labeled olfactory epithelial cells that had been pre-mixed in different ratios were sorted on the device. For each sample, 100 cells were sorted, and cells collected at the recovery well were tabulated. An accuracy of close to 100% was observed (Table 1).

**Table 1:** Sorting efficiency and cell recovery from different input conditions.

	Positive collection well		Negative collection well		
Mix ratio	Fluo4 (+)	Fluo4 (−)	Fluo4 (+)	Fluo4 (−)	Cells sorted
50-50	47	0	0	53	100
30-70	20	1	0	79	100
10-90	7	0	0	93	100

### Ionophore and L-lysine stimulations

**Figure 3:**
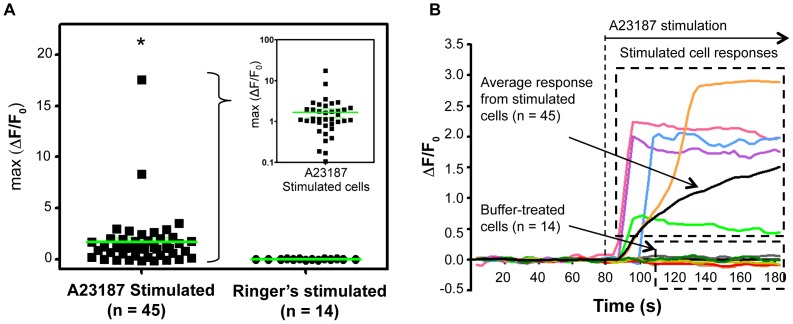
Ionophore-induced calcium influx in cells extracted from zebrafish olfactory epithelium. (A) Maximum fluorescence change in individual cells after stimulation with A23187 (n = 45) or Ringer's (n = 14). The inset depicts the data for the same cells on a log scale. (*p = 0.0144, unpaired one-tailed Student's *t*-test). (B) Change in fluorescence intensity relative to baseline fluorescence intensity (ΔF/F_0_) plotted over time for representative cells. The black trace indicates the averaged response. A23187 was injected into the cell trap at the indicated time (80 s), and remained in the trap. Control cells stimulated with Ringer's buffer did not exhibit a significant change in fluorescence intensity.

Our aim in designing the device was to identify a subset of cells within a heterogeneous population that responded to a specific stimulus, using increase in intracellular calcium levels as a measure of the cellular response, and to recover both responsive and non-responsive cells for molecular characterization. As a proof-of-concept experiment, we assayed calcium influx in cells isolated from the zebrafish olfactory epithelium in response to treatment with the ionophore A23187, which promotes the rapid diffusion of calcium across biological membranes [37]. Freshly isolated cells suspended in Ringer's buffer, containing 1.8 mM CaCl_2_, were labeled with a fluorescent calcium indicator (Fluo 4-AM) and loaded into the device. Single cells were captured in the trap and exposed to 5 µM A23187 for 15 s. Fluorescence images were taken every 3 seconds beginning 1 minute before the stimulation event and extending to 5 minutes after start of stimulation. From a total of 45 randomly selected cells, the maximal fluorescence increase in response to the ionophore was, on average, 1.8 fold (Figure 3A), consistent with other studies using A23187 and similar concentrations of calcium in the cell suspension buffer [38]. Although responses were heterogeneous, in terms of latency and magnitude, most cells reached a 50% increase in fluorescence intensity within the first 15 s of exposure to A23187 (Figure 3B). As a control, we stimulated a number of isolated cells with Ringer's buffer. No significant change in fluorescence intensity was observed in these cells (Figure 3A, B). These data establish the feasibility of using the device to screen cells for changes in intracellular calcium levels in response to a stimulus. They also establish that even though cells are in a suspension, fluorescence changes due to ligand introduction can be distinguished from any change that may occur simply by the introduction of a reagent such as Ringer's buffer.

**Figure 4:**
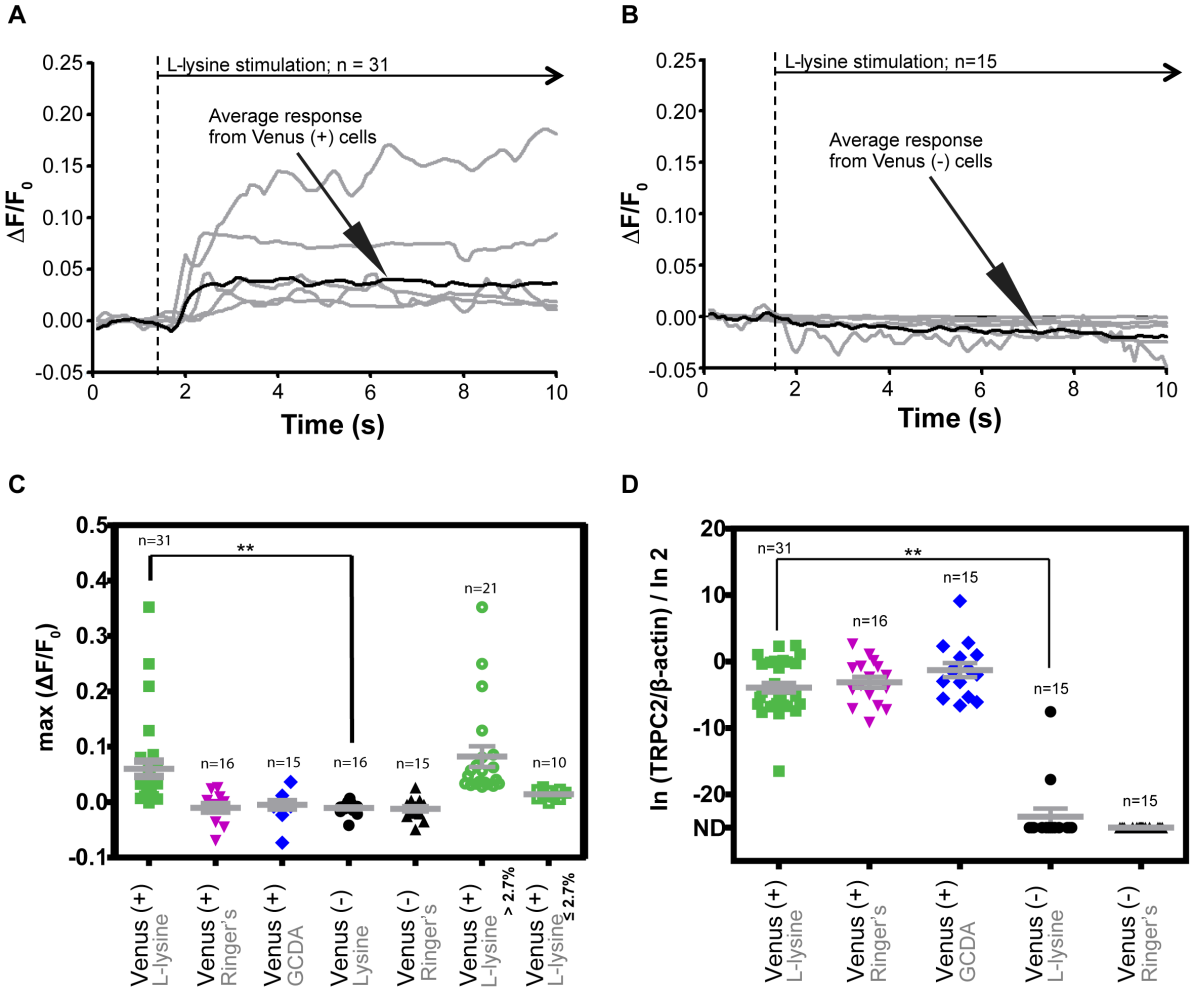
Lysine stimulation of a mixed cell population from the olfactory epithelium of TRPC2:Venus transgenic zebrafish. (A) Relative change of fluorescence intensity in cells expressing Venus, following stimulation with L-lysine. Gray lines represent the response of individual cells while the black line plots the averaged response. L-lysine was injected into the cell trap at the time indicated by the dashed line. (B) Relative fluorescence intensity change in cells with no observable Venus fluorescence, in response to L-lysine. (C) Maximum fluorescence intensity change measured for each cell after stimulation with the indicated ligand. Venus expressing cells (labeled Venus (+)) show a response to L-lysine, but not to Ringer's or GCDA. Cells that did not express Venus (labeled Venus (−)) did not respond to L-lysine or to Ringer's. For clarity, the last two columns re-plot the data for Venus-expressing cells stimulated with L-lysine, grouping the data points based on whether the maximum fluorescence change for a cell was above or below the threshold of 2.7%. (D) qRT-PCR analysis of cells in panel (C), showing relative abundance of *TRPC2* and *β-Actin* mRNA. All Venus expressing cells expressed relatively high levels of *TRPC2*. *TRPC2* mRNA was detected in only two cells that did not express Venus. ND: not detected. The corresponding data for OMP, EF1α, and B2M are shown in Figures S3, S4, and S5 in File S1. [(C): **p = 0.0005; (D): **p<0.0001; unpaired one-tailed Student's *t*-test)].

We utilized the device to identify and molecularly characterize a subpopulation of olfactory sensory neurons (OSNs) that respond to a specific odor cue, L-lysine, from the heterogeneous population of cells in the zebrafish olfactory epithelium. In general, amino acids are sensed by neurons that express the channel TRPC2 [32], [39], [40], and these are labeled in the TRPC2:Venus transgenic line [32]. In contrast, bile acids are sensed by OSNs that express the olfactory marker protein (OMP). We loaded cells from the olfactory epithelium of TRPC2:Venus fish onto the device and monitored the change in intracellular calcium levels as individual cells were exposed to one of three conditions: 10 µM L-lysine, Ringer's buffer alone, or 10 µM of the bile acid glycochenodeoxycholic acid (GCDA). Rhod 2-AM was used as a calcium indicator here to avoid overlap with the fluorescence of Venus. Based on the distribution of changes in fluorescence intensity (ΔF/F_0_) observed when cells were stimulated with buffer alone, we set the threshold for scoring a positive response to L-lysine or GCDA as ΔF/F_0_>2.7%, which is similar to the threshold value used in previous studies [39], [41], [42]. A subset of the cells that expressed Venus (68%) were scored as responsive to L-lysine (Figure 4A, C). In contrast, no significant increase in fluorescence intensity in response to L-lysine was observed among the cells that did not express Venus (Figure 4B, C). None of the Venus-expressing cells tested responded to GCDA (Figure 4C).

After screening each cell by fluorescence microscopy and recovering the cell in one of the recovery wells on the device, we manually transferred the cell to a PCR tube for cell lysis. We collected cells from three independent experiments for mRNA extraction and qRT-PCR. For this pilot experiment, we quantified the relative abundance of five genes: TRPC2, OMP, and the housekeeping genes EF1α, B2M, and ß-Actin. As expected, all of the cells that responded to L-lysine expressed detectable levels of TRPC2 mRNA (Figure 4D). TRPC2 mRNA was undetectable in all Venus (−) cells, with the exception of two cells, which had TRPC2 mRNA levels comparable to the Venus-expressing cells. These cells may represent a subtype in which transcription from the endogenous TRPC2 is activated via cis-acting sequences that are missing from the promoter fragment used to drive expression of the TRPC2:Venus reporter gene.

Consistent with previous reports that expression of TRPC2 and OMP in OSNs is generally mutually exclusive [32], we observed a sizeable fraction of OMP-expressing cells among the cells that did not express Venus (Figure S3 in File S1). There were, however, six cells that expressed both TRPC2 and OMP mRNA, five of which also expressed TRPC2:Venus (as determined by fluorescence, Figure S3 in File S1). Thus, there may be a small subset of OSNs in the zebrafish olfactory epithelium that express both TRPC2 and OMP.

## Discussion

We have developed a microfluidic device to interrogate single cells that allows recording of dynamic responses to a ligand, followed by sorting and enrichment of a desired subpopulation. This was demonstrated here using olfactory sensory neurons of the zebrafish, and monitoring their response to the odorant L-lysine. Integrated microfluidic systems offer several advantages for single cell analysis. The micro-scale size of such devices ensures precise fluid control due to laminar flow, requires significantly lower volumes of reagents compared with standard formats, and provides a platform where tedious experimental protocols can be automated to reduce associated human errors. Furthermore, these devices incur minimal costs and can be replaced for each experiment, which reduces chances of cross-contamination.

In order to achieve a gentle yet efficient sorting process, we have incorporated different components made up of micro-valves. The device is capable of reliable stimulus delivery and performing consistent cell recovery. Dynamic changes in the cells after stimulation are actively monitored via time-lapse fluorescence microscopy. This creates the possibility of working with small number of input cells and is beneficial for sensitive measurements in situations such as biopsies and rare cell samples [43]. We have also ascertained that cell viability is unaffected during or after the entire process. Screening cells based on their dynamic properties by this method was low-throughput (approximately 30 seconds for stimulating and sorting) to ensure the reliability of sorting single cells with minimum error. However, it may be possible to increase throughput by parallelization of the cell trap to allow simultaneous interrogation of multiple cells, as well as automating ligand delivery, image analysis and cell sorting. In this case, real-time image analysis can be used to determine when cells enter the trap, as well as to determine their response upon stimulus introduction, as this would be reflected by a change in fluorescence intensity.

## Supporting Information

File S1 **This contains all the supplementary figures. Figure S1, Summary of device fabrication using soft lithography. Figure S2, Schematic of the pneumatic connections for operating the device. Figure S3, Relative OMP and β-Actin mRNA abundance for the cells shown in Fig. 5.** ND: Not detected. **Figure S4, Relative EF1α and β-Actin mRNA abundance for the cells shown in Fig. 5.** ND: Not detected. **Figure S5, Relative B2M and β-Actin mRNA abundance for the cells shown in Fig. 5.** ND: Not detected. **Table S1, Primer sequences used in qRT-PCR. Table S2, Effects of input concentration on single cell trapping.**
(DOCX)

File S2 **AutoCAD file used to create the photo mask.**
(DWG)

File S3 **A .exe file used to operate the device.** The GUI allows control of valves, and hence movement of cells and delivery of stimulus or buffer to cells.
(EXE)
