# Supplementary material for: A Microfluidic Device to Sort Cells Based on Dynamic Response to a Stimulus
Source: PLoS One. 2013 Nov 8;8(11):e78261. doi: 10.1371/journal.pone.0078261 (PMC3826715; doi:10.1371/journal.pone.0078261)
Supplement: File S1 — This contains all the supplementary figures. Figure S1, Summary of device fabrication using soft lithography. Figure S2, Schematic of the pneumatic connections for operating the device. Figure S3, Relative OMP and β-Actin mRNA abundance for the cells shown in Fig. 5. ND: Not detected. Figure S4, Relative EF1α and β-Actin mRNA abundance for the cells shown in Fig. 5. ND: Not detected. Figure S5, Relative B2M and β-Actin mRNA abundance for the cells shown in Fig. 5. ND: Not detected. Table S1, Primer sequences used in qRT-PCR. Table S2, Effects of input concentration on single cell trapping. (DOCX) [file pone.0078261.s001.docx]

**Supplemental information**

**File S1, consisting of:**

**Table S1.** Primer sequences used in qRT-PCR.

**Table S2.** Effects of input concentration on single cell trapping.

**Figure S1.** Summary of device fabrication using soft lithography.

**Figure S2.** Schematic of the pneumatic connections for operating the device.

**Figure S3.** Relative OMP and β-Actin mRNA abundance for the cells shown in Fig. 5.

**Figure S4.** Relative EF1α and β-Actin mRNA abundance for the cells shown in Fig. 5.

**Figure S5.** Relative B2M and β-Actin mRNA abundance for the cells shown in Fig. 5.

**File S2.** Auto-CAD file

**File S3.** LabView scripts for operating the device.

**Table S1.** Primer sequences used in qRT-PCR

| **Gene Name** | **RefSeq ID** | **Gene symbol** | **Primer Sequence (5’ → 3’)** |
| --- | --- | --- | --- |
| Beta-actin | NC_  007114.5 | β -actin | (F) CGAGCTGTCTTCCCATCCA  (R) TCACCAACGTAGCTGTCTTTCTG |
| Transient receptor potential cation channel, subfamily C, member 2 | NM_  001030166 | TRPC2 | (F) TCCTGAACAGCTGTGTGGAGATCG  (R) TCAAGGTCTGGTGGTCCAGTTC |
| Olfactory marker protein B | NM_  173281 | OmpB | (F) CATTTCACCCGCTGGAACATTCG  (R) CTGGGAAGTGGCGATGATGTTTAG |
| Elongation factor 1 alpha | NC_  007130.5 | EF1α | (F) CTGGAGGCCAGCTCAAACAT  (R) ATCAAGAAGAGTAGTACCGCTAGCATTAC |
| Beta 2 microglobulin | NM_  131163 | B2M | (F) GATTGTCTGCTTGGCTCTCTCG  (R) ACACATGAACTTTCGGAGTGGAG |

**Table S2.** Effects of input concentration on single cell trapping

| Input bead concentration  (beads / ml) | Number of beads per min | Probability of single  cell in trap |
| --- | --- | --- |
| 20×10^6^ | 95 | 50% |
| 2×10^6^ | 53 | 98% |
| 2×10^5^ | 27 | 100% |
| 2×10^4^ | 4 | 100% |
| 2×10^3^ | 0-1 | 100% |

Peristaltic pump setting: 1.67Hz. Beads were suspended in 1x PBS.


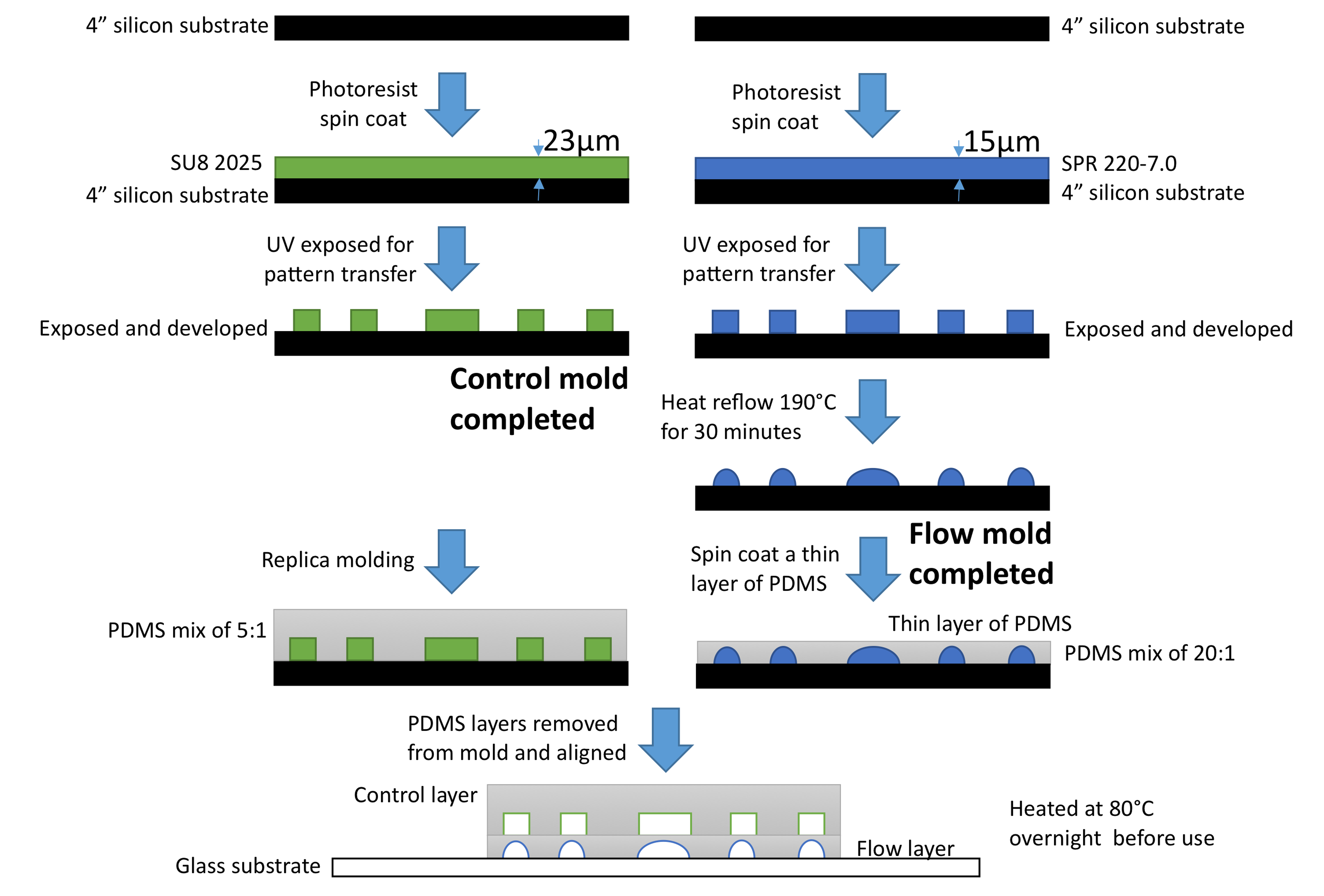


**Figure S1.** Summary of device fabrication using soft lithography.


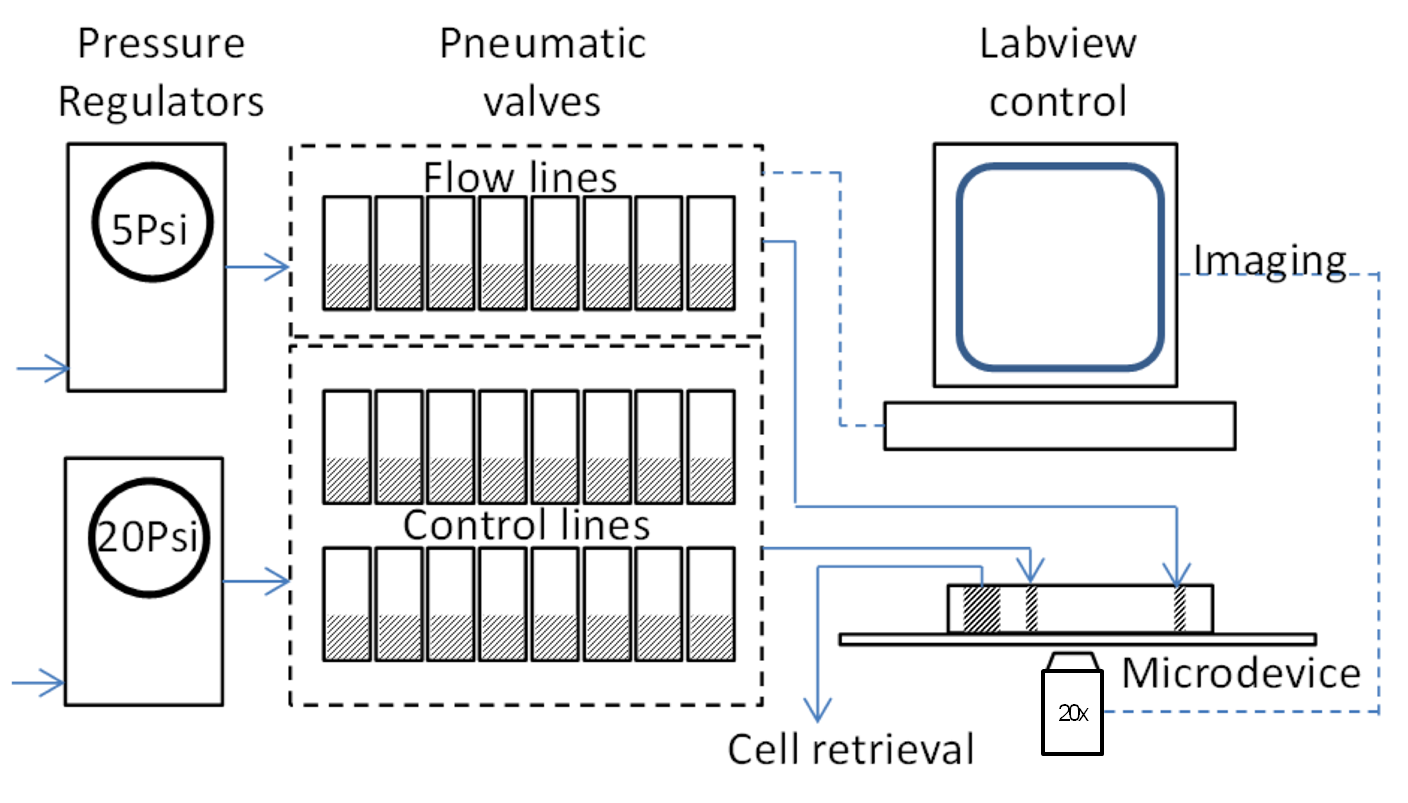


**Figure S2.** Schematic of the pneumatic connections for operating the device.

n=31

n=15

n=15

**TRPC2:gap-YFP cells GCA stimulus**

**YFP (-) cells GCA stimulus**

**ln (OMP/β-actin) / ln 2**

**TRPC2:gap-YFP cells Ringer’s stimulus**

**TRPC2:gap-YFP cells Lysine stimulus**

**YFP (-) cells**

**Lysine stimulus**

n=15

n=16


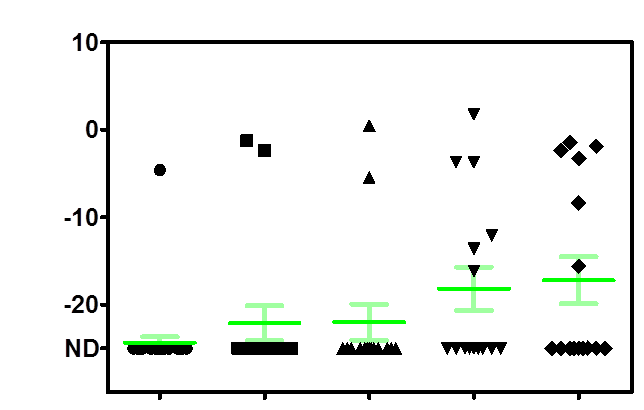


**Figure S3.** Relative OMP and β-Actin mRNA abundance for the cells shown in Fig. 5. ND: Not detected.

**TRPC2:gap-YFP cells**

**Lysine stimulus**

**YFP (-) cells**

**Lysine stimulus**

**ln (EF1-α/β-actin) / ln 2**


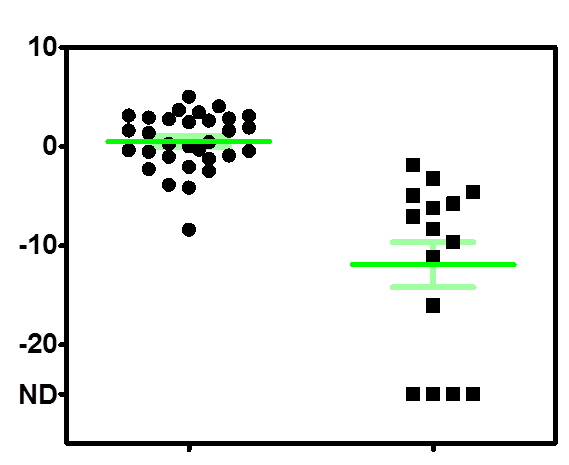


**Figure S4.** Relative EF1α and β-Actin mRNA abundance for the cells shown in Fig. 5. ND: Not detected.

**TRPC2:gap-YFP cells**

**Lysine stimulus**

**YFP (-) cells**

**Lysine stimulus**

**ln (B2M/β-actin) / ln 2**


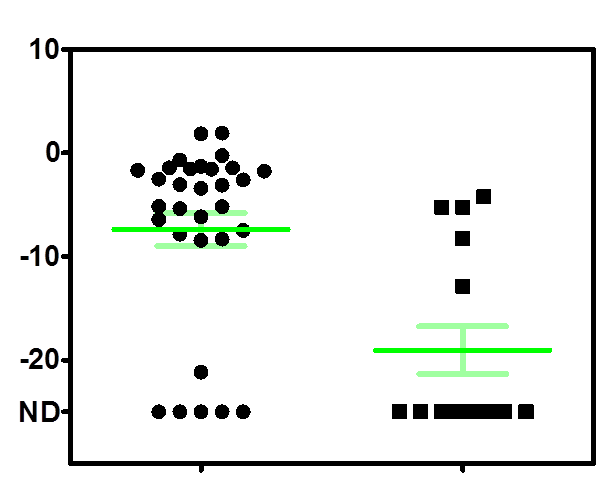


**Figure S5.** Relative B2M and β-Actin mRNA abundance for the cells shown in Fig. 5. ND: Not detected.
